# Early screening tool for developmental delay in infancy: Quantified assessment of movement asymmetry using IR-UWB radar

**DOI:** 10.3389/fped.2022.731534

**Published:** 2022-10-14

**Authors:** Jae Yoon Na, Won Hyuk Lee, Young-Hyo Lim, Seok Hyun Cho, Sung Ho Cho, Hyun-Kyung Park

**Affiliations:** ^1^ Department of Pediatrics, Hanyang University College of Medicine, Seoul, South Korea; ^2^ Department of Electronics and Computer Engineering, Hanyang University, Seoul, South Korea; ^3^ Division of Cardiology, Department of Internal Medicine, Hanyang University College of Medicine, Seoul, South Korea; ^4^ Department of Otorhinolaryngology-Head and Neck Surgery, Hanyang University College of Medicine, Seoul, South Korea

**Keywords:** IR-UWB radar sensor, quantification of movement asymmetry, actigraphy, early screening tool for developmental delay, cerebral palsy, preterm, hydrocephalus

## Abstract

In the untact COVID-19 era, the feasibility of a noncontact, impulse-radio ultrawideband (IR-UWB) radar sensor has important medical implications. Premature birth is a major risk factor for brain injury and developmental delay; therefore, early intervention is crucial for potentially achieving better developmental outcomes. Early detection and screening tests in infancy are limited to the quantification of differences between normal and spastic movements. This study investigated the quantified asymmetry in the general movements of an infant with hydrocephalus and proposes IR-UWB radar as a novel, early screening tool for developmental delay. To support this state-of-the-art technology, data from actigraphy and video camcorder recordings were adopted simultaneously to compare relevant time series as the infant grew. The data from the three different methods were highly concordant; specifically, the *ρ_z_* values comparing radar and actigraphy, which served as the reference for measuring movements, showed excellent agreement, with values of 0.66 on the left and 0.56 on the right. The total amount of movement measured by radar over time increased overall; movements were almost dominant on the left at first (75.2% of total movements), but following shunt surgery, the frequency of movement on both sides was similar (54.8% of total movements). As the hydrocephalus improved, the lateralization of movement on radar began to coincide with the clinical features. These results support the important complementary role of this radar system in predicting motor disorders very early in life.

## Introduction

In recent years, the incidence of preterm births has grown considerably, and despite impressive technological improvements in the equipment used in neonatal intensive care units (NICUs), premature infants remain at increased risk for conditions responsible for neurodevelopmental delays, such as cerebral palsy and cognitive deficits. If the initiation of intervention is delayed, an important brain plasticity window in preterm infants, in which targeted therapy could prevent negative neurological outcomes, may be missed. Therefore, earlier screening and identification of signs of neurodevelopmental delays is crucial (1).

Impulse radio ultrawideband (IR-UWB) radar sensors are high-precision electromagnetic devices that can recognize the motion of an object at a distance. IR-UWB radar has various advantages in medical applications, such as its contactless/wireless capability, high data processing rate, low radiation exposure risk for the human body, and convenience for daily use in and out of the hospital. Recently, we introduced a signal model for IR-UWB radar and an algorithm for activity measurement and quantification in movement disorders (2). We later showed that this technology was capable of capturing vital signals such as heart rates and respiration even in very small preterm infants (3, 4). This radar is a state-of-the-art sensing device that can be used appropriately in the COVID-19 era and digital healthcare markets.

Existing comprehensive developmental tests for screening in infancy, such as the Bayley Scales of Infant and Toddler Development (Bayley-III) and the Test of Infant Motor Performance (TIMP), have low compliance rates and are dependent on subjective rating scales completed by parents and brief observations by doctors in outpatient clinics. Therefore, their results are often obscure and have low specificity (5). Additionally, these tests require specialized training for administration and prolonged handling that can be detrimental to fragile preterm infants (1). Numerous studies have investigated objective measurements to predict and detect motor disability at a very early age (so-called “find-early-and-intervene-early” paradigms) using video recording or direct observations, motion tracking sensors, actigraphy, and neurofunctional biomarkers to capture abnormal spontaneous general movements, spasticity, rigidity, and weakness. These findings have been limited to reviews of movements over only 30 min to 3 h (6, 7).

To date, both actigraphy and video camcorder analyses, which are standard methods for detecting movements, as well as methods using machine learning (5) and wearable sensors (8) have been the subject of certain investigations. Despite the substantial literature and the fact that this early developmental period may represent an important timeframe for studying developmental disorders, these trials have historically received little research and clinical attention.

Based on our experience in measuring small movements such as breathing and heart rate in neonates using IR-UWB radar (9, 10), we believe that this noncontact sensing technology can be used to identify abnormal movement and allow their early detection and quantification, especially while the infant is growing and her neuropsychomotor development is not fully complete. Agreement with clinical findings of motor disability can be improved through long-term radar data collection, as it is easily applicable in a contactless manner to the infant.

The objective of this study was to investigate the feasibility of IR-UWB radar as a contactless, early screening tool to predict and detect developmental delays and the likelihood of transition to cerebral palsy and to quantify movement asymmetry from the neonatal period to infancy.

## Materials and methods

The study protocol adhered to the Declaration of Helsinki and was approved by the Institutional Review Board of Hanyang University Medical Centre (No. 2017-09-046-002). Written informed consent was obtained from the parents of the patient.

### Subject

The patient was a preterm infant who was born at 25 + 5 weeks of gestation and weighed 840 grams at birth. Germinal matrix hemorrhage was observed on brain ultrasonography (US) performed one week after birth. The brain hemorrhage gradually progressed to obstructive hydrocephalus in both ventricles, mainly on the right side. To control the infant's increased intracranial pressure (IICP), several external ventricular drains (EVDs) and ventriculoperitoneal (VP) shunts were implanted. The first experiment was performed at 19 days of corrected age (4 months after birth), one month after the insertion of two EVDs into the right ventricle and the first VP shunt in the left ventricle. Two days after the first experiment, a third EVD and a second VP shunt were inserted into the right ventricle. The experiment described in the next subsection was performed on days 67, 102, and 152 of corrected age (5 months 2 weeks, 6 months 3 weeks, and 8 months 2 weeks after birth, respectively) alongside serial brain US and CT.

### Experimental setup

**Figure 1:**
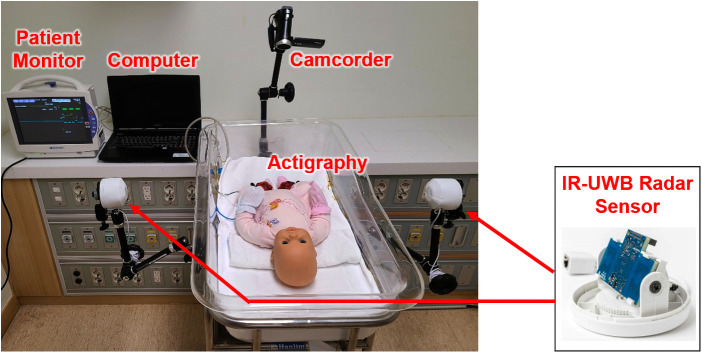
Experimental environment of the IR-UWB radar sensor. The two IR-UWB radar sensors (width × depth × height: 5.8 × 3.4 ×  1.8 cm^3^; weight: 150 g; actual sensor chip: 2.2 × 1.2 × 0.6 cm^3^, 18 g) are covered with a white cap for clinical application to monitor the patient and then mounted at the end of specially designed arms and placed one on the right and one on the left side of the infant.

Two IR-UWB radar sensors (XK300-MVI, Xandar Kardian, Toronto, ON, Canada) were placed with a tripod arm on both sides of the infant for quantitative measurement of the infant's left- and right-sided movements. The radars were placed approximately 30 cm away from the infant's cradle bilaterally, with the radar module oriented toward the infant. The experiment was conducted in the NICU and general ward at Hanyang University Hospital, Seoul, Korea. Pediatricians, neonatal nurses, and researchers observed the entirety of the experimental procedure. To monitor vital signs during the experiment, electrocardiography (ECG) and saturation monitoring sensors were attached to the infant's skin (Figure 1). Each experiment was performed from 30 min to 2 h, and when the help of the parents, doctors or nurses was needed, any data collected were excluded from the analysis.

### Radar system protocol

The center frequency of the IR-UWB radar sensor is 8.748 GHz, the radiation power is 68.85 µW, and the received signal sampling rate is 23.328 GS/s. Here, the radar devices monitored the infant's movement 20 times per second and transmitted it to a PC over USB. The received signals were calculated as quantified movements through signal processing in MATLAB. The operating system was Windows 10.

To quantify infant movement using the two IR-UWB radar sensors, the distance information from the sensor to the center of the infant body is needed. Since signals received from each IR-UWB radar sensor reflect the movement of the entire infant’s body, the distance from the two sensors to the center of the infant's body had to be known first to quantify the movement and separate it into movement of the left and right sides of the infant's body. To find the distance from each radar sensor to the center of the newborn's body, it was necessary to find the point of greatest respiratory movement in the newborn’s body; this point corresponds to the infant's abdomen or chest, and the distance to that point is the distance to the center of the body. In a previous study, we suggested a way to find the point where the movement was most active by measuring vital signs, including breathing and heart rate (3). This method allows the calculation of the distance from the IR-UWB radar sensors to the center of the abdomen during experimental data collection even without knowing the exact distance between the installed IR-UWB radar sensors or the exact location where the newborn is placed.

$$\begin{array}{rl} E_{i}[n]= & \;\overset{L_{\mathrm{signal}}}{\underset{k=0}{\sum }}g_{i,n}[k],\\ g_{i,n}=\; & \begin{array}{c} |y_{i,n}[k]-y_{i,n-1}[k]|\;\;\mathrm{if}\;y_{i,n}[k]>T_{i}[k]\\ 0\mathrm{if}\;y_{i,n}[k]\leq T_{i}[k] \end{array} \end{array}$$

**Figure 2:**
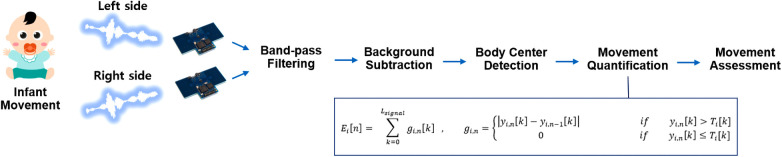
Algorithm for movement assessment using radar signals. The signals received from the two IR-UWB radar sensors are bandpass filtered to reduce the level of noise, and background subtraction is used for clutter signal removal. These preprocessed signals are the used to quantify the movement of the left and right sides of the infant through digital signal processing.

The $L_{\mathrm{Signal}}$ used in the equation refers to the signal index at the point corresponding to the distance from each IR-UWB radar sensor to the center of the infant's body, and $y_{i,n}[k]$ is the signal from the radar. If motion does not occur over a certain length of time, the values of the *k*-th sample of the *i* -th radar will always be constant. However, when motion occurs within the radar observation range, the corresponding $y_{i,n}[k]$ will look different from the previous signal $y_{i,n}[k]$, and the probability characteristics will be much different from those of the signal when there is no movement. Thus, movement can be quantified using the sum of the difference between two consecutive signals. However, noise from the radar signals cannot be reduced simply by quantifying movement through differences in consecutive signals. Therefore, we only used samples above a threshold $T_{i}[k]$ from the received signals to account for noise. When a doctor or nurse approached the cradle for clinical treatment, any movements that occurred at that time were excluded from the experimental data because their movements were also included in the radar signal. Since the radar monitored the infant's movements 20 times a second, $E_{i}[n]$ represents 20 results per second; therefore, the average of these 20 values was calculated for statistical analysis. The algorithm for movement assessment using radar signals is shown in Figure 2.

### Conventional measurements as the reference

The experimental procedures were also recorded with a video camcorder (Digital HD Video Camera Recorder, Sony Corporation, China) to serve as the reference data. Two actigraphy sensors (xGT3X-BT, Actigraph, Florida, US) were attached to each ankle using a string to monitor the rest/activity cycle. The actigraphy sensors each possess an accelerometer that measures the activity of the body parts to which they are attached. The movements measured from these sensors were extracted as the vector magnitude value through ActiLife (Actigraph, Florida, Us) software. The actigraphy data were sampled every second, and the sampling rate was scaled to allow comparison with the radar data. The movements were also measured using computer vision tracking by analyzing the video camcorder (VT by video) recordings and the radar and actigraphy data. We performed vision tracking using a modified algorithm called the Continuously Adaptive Mean Shift (CAMSHIFT) algorithm (11, 12); further details on the vision tracking algorithm can be found in the Supplementary Methods.

### Statistical analysis

The left and right movements measured by the IR-UWB radar and actigraphy sensors and VT by video were assessed by agreement using Fisher's z-transformation (13, 14). The concordance correlation coefficients (CCCs) were expressed in *ρ_z_*, and 95% confidence intervals for the CCC were also suggested.

## Results

### Comparison among IR-UWB radar, actigraphy, and video tracking activity

**Figure 3:**
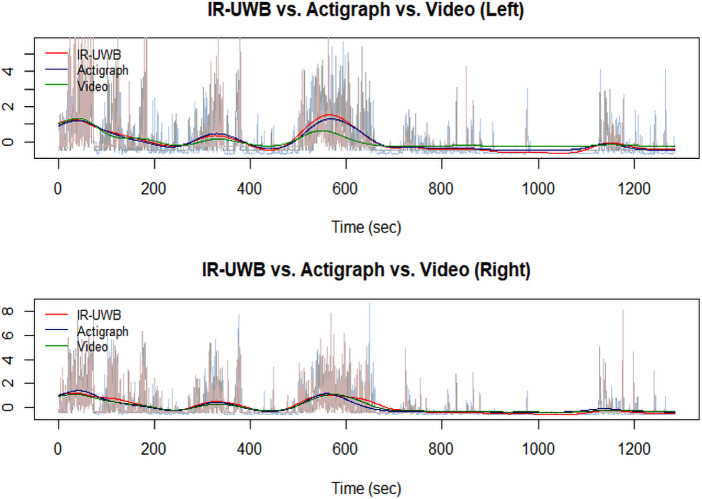
Representative results of the quantified movement waveforms from IR-UWB radar (red), actigraphy (blue), and video (green). Quantified movements on the left and right sides of the infant were obtained simultaneously by each sensor. The IR-UWB radar sensor signal correlates well with the video and actigraphy sensor signals.

The data from the three different observation methods were concordant (Figure 3 and Table 1). First, for the movements measured from the left, the IR-UWB radar and actigraphy achieved a *ρ_z_* of 0.6646, while the IR-UWB radar and VT by video achieved a *ρ_z_* of 0.4391. For the movements from the right, the *ρ_z_* between IR-UWB radar and actigraphy was 0.5636, and that between IR-UWB radar and VT by video was 0.5707.

**Table 1:** Concordance correlation coefficients between the two methods in the third experiment.

Device	Side	*ρ_z_* ^a^	95% CI^b^
IR-UWB vs. Actigraphy	Left	0.6646	[0.6450, 0.6842]
	Right	0.5636	[0.5466, 0.5805]
IR-UWB vs. Video	Left	0.4391	[0.4191, 0.4591]
	Right	0.5707	[0.5505, 0.5910]
Actigraphy vs. Video	Left	0.3945	[0.3637, 0.4255]
	Right	0.3900	[0.3726, 0.4073]

^a^ *ρ_z_* is the correlation coefficient normally approximated by Fisher's transformation.

^b^ 95% CI is the 95% confidence interval for the correlation coefficient.

### Detection of serial neurobehavioral changes

**Figure 4:**
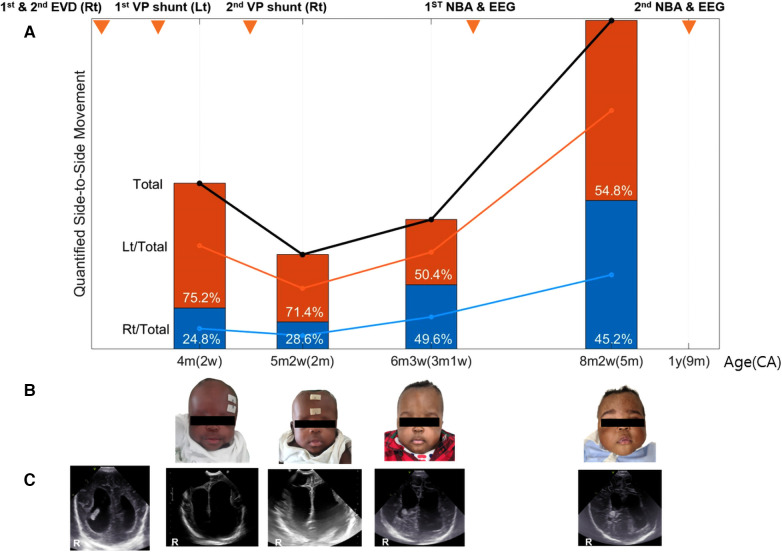
Quantification and serial trends in side-to-side movement asymmetry. (**A**) Quantified movement measurements and clinical courses. At the first two time points, left-side motion was dominant (left-side vs. right-side, 75.2% vs. 24.8%), but over time, the movements of the two sides achieved greater symmetry. (**B,C**) Changes in anterior fontanelle and head ultrasonography. From the beginning, encephalomalacic changes in the right brain were observed. Over time, the asymmetry of the infant's head and ventricular dilatation, observed on brain ultrasonography, improved through repeated surgical procedures. The differences in movement between the two sides cannot be well recognized by conventional evaluation methods. CA, corrected age; Lt/Total (%), ratio of quantified left-dominant movement to total movement; Rt/Total (%), ratio of quantified right-dominant movement to total movement; NBA, neurobehavioral assessment; EEG, electroencephalogram.

Initially, the movement was primarily dominant on the left, but over time, the frequency of movements on both sides became similar (Figure 4).

The assessments for the diagnosis of neurodevelopmental disorders were conducted at 4 months 1 week of corrected age and 9 months of corrected age. All assessments were performed by experienced rehabilitation specialists and pediatric neurologists. First, the infant demonstrated greater muscle tone in the lower extremities than in the upper extremities on physical examination. In particular, the left lower extremity and Achilles tendon had a Modified Ashworth Scale (MAS) grade of 3, indicating greater muscle tone than the right extremity and tendon, which achieved a MAS grade of 2. On sleep electroencephalography (EEG) performed on the same day, no definite seizure waves were observed. Overall, a normal EEG pattern was observed, but in part, mild asymmetry between both hemispheres was shown. Relatively poor anterior-posterior gradients and mild voltage depression of the right hemisphere were noted, suggesting a signal of right cerebral dysfunction. The infant was evaluated with four rehabilitation tests (Erhardt Developmental Prehension Assessment, EDPA; Pediatric Evaluation of Disability Inventory, PEDI; Denver Developmental Screening Test II, DDST-II; Gross Motor Function Classification System, GMFCS) commonly used for children. However, at the time of the tests, the infant was too young to provide accurate results for determining the possibility of movement asymmetry. Moreover, none of the above four methods analyzes the differences between left- and right-sided movements.

## Discussion

This study implemented a longitudinal design to follow the general movements of an infant who was born as an extremely low birth weight infant (ELBW) and later developed unilateral hydrocephalus. The contactless IR-UWB radar sensor continuously assessed and quantified the infant's side-to-side movements, and its results agreed well with the actigraphy and video recording data. Furthermore, the radar sensor system enables physicians to detect and predict neurodevelopmental aggravation or improvement earlier as a complement to brain imaging and neurobehavioral assessment.

Despite remarkable improvements in the survival of preterm infants, they remain at an increased risk for long-term neurodevelopmental outcomes (15). A large cohort study suggested that children with ELBW and severe intraventricular hemorrhage (IVH) requiring shunt insertion are at the greatest risk for adverse neurodevelopmental and growth outcomes at 18 to 22 months (16). Although the optimal time for the most accurate analysis of general movements is debated and the process of early screening for developmental delays remains difficult, various analyses of video recordings of general movements, serial imaging, and neurobehavioral screening tests have reported them as feasible early predictors of neurological deficits for identifying at-risk infants and establishing eligibility for intervention (17–19). However, existing tests have low compliance rates, are inconvenient given the need to visit a general hospital, require sedation of the infant, lack certified and trained specialists, and involve time constraints for long-term observation and prolonged handling that can be detrimental to fragile neonates. In particular, preterm infants are vulnerable to the stressful physiological effects of neurodevelopmental testing (1). A recent machine learning model for computer-based infant movement assessment using video recordings showed high sensitivity (92.7%) and relatively low specificity (81.6%).

The high-precision electromagnetic IR-UWB radar sensor used in this study is a noninvasive, contactless, wireless, and continuous diagnostic technology for monitoring and quantifying abnormal infant movements. Moreover, the radar system has many advantages, such as its ubiquitous availability even with limited healthcare resources, convenience in daily use in and out of the hospital, inexpensiveness, high data-processing rate, and long-term applicability for predicting neurological deficits. Recently, we introduced a signal model for the IR-UWB radar system and an algorithm for activity measurement (2, 20). However, that study measured notable movements in older children, and its current application in a small infant with hydrocephalus was challenging and the first such report to our knowledge. This remote monitoring system may be suitable as an early screening tool for improving developmental outcomes and avoiding diagnostic delay, particularly in the COVID-19 era, as an untact medical concept. Early detection for groups at high risk of developmental delays would improve access to community services and well-being for parents, provide economic support for families in need of care and facilitate earlier management (21–23).

The longitudinal data from the IR-UWB radar revealed the first asymmetries at the age of 2–4 months and marked asymmetrical movement in subsequently developed hemiplegia contralateral to the side of the lesion. Although obstructive hydrocephalus and encephalomalacic changes in the right brain have been observed from the beginning, most neurobehavioral examinations might still yield normal results (24). Only a careful examination by a rehabilitation expert and minor changes on EEG could be used to detect asymmetry on both sides. As the infant's hydrocephalus improved after surgery, the quantified discrepancy in the side-to-side movements decreased. Moreover, we demonstrated that the radar was not inferior to actigraphy and image analysis.

This quantification of infant movement through IR-UWB radar has certain limitations. This is a novel technology, but only a small number of experiments have been performed on a single patient. Second, the radar cannot be used for older infants and children to achieve a “find-early-and-intervene-early” outcome because of confusion in the data from normal developmental movements such as rolling over and crawling. Third, the number of radars used at the same time remains excessive and should be reduced for easier use.

In summary, we quantified the preoperative and postoperative trends in movement asymmetry in an infant with hydrocephalus using contactless, noninvasive, and continuous IR-UWB radar sensors, although the majority of neurobehavioral assessments appeared to be similar in the side-to-side comparisons. Moreover, these results corresponded well with those of other assessments, including actigraphy and video recordings. This report focused on a single infant, but we followed up the serial trend in movements at bedside and with minimal interference with standard clinical practices. We believe this sensor system can be a clinically feasible alternative to general observational movement assessments as an early prediction tool for neurodevelopmental delays and could encourage further validation in clinical settings.

## Data Availability

The raw data supporting the conclusions of this article will be made available by the authors, without undue reservation.
