# Supplementary material for: Early screening tool for developmental delay in infancy: Quantified assessment of movement asymmetry using IR-UWB radar
Source: Front Pediatr. 2022 Oct 14;10:731534. doi: 10.3389/fped.2022.731534 (PMC9614076; doi:10.3389/fped.2022.731534)
Supplement: Supplementary file 1 [file DataSheet1.docx]

**Supplementary Methods: Video Tracking Activity**

The Continuously Adaptive Mean Shift (CAMSHIFT) algorithm is used for tracking in the field of computer vision with relatively simple calculations and dynamically changing color probability distributions derived from video frame sequences. Generally, to establish an initial location and compute a probability distribution for the tracked object, a region of interest (ROI) is determined based on the HSV color space. In our case, after placing a red glove and sock on the right hand and foot and a blue glove and sock on the left hand and foot, image analysis was performed using this color information. Utilizing the probability distribution, the centroid of the ROI was computed and moved toward the highest density location in the distribution until the information converged. The size and position of the new ROI were mathematically regulated, and then the whole procedure was repeated.
